# Patterns of thromboembolic pulmonary vascular disease in COVID-19

**DOI:** 10.1177/2045894020979198

**Published:** 2021-01-21

**Authors:** Yousef Shahin, Smitha Rajaram, Vivak Parkash, James M Wild, David G Kiely, Andrew J Swift

**Affiliations:** 1 Department of Infection, Immunity and Cardiovascular Disease, University of Sheffield, Sheffield, UK; 2 Department of Clinical Radiology, Sheffield Teaching Hospitals, Sheffield, UK; 3 INSIGNEO, Institute for In Silico Medicine, University of Sheffield, UK; 4 Sheffield Pulmonary Vascular Disease Unit, Royal Hallamshire Hospital, Sheffield, UK

**Keywords:** coronavirus, CT-LSIM, iodine subtraction mapping, pulmonary embolism, microvascular thrombosis

## Abstract

SARS-CoV-2 (COVID-19) is associated with increased thrombosis. Here, we demonstrate patterns of pulmonary vascular disease in COVID-19 including classical acute pulmonary embolism and subsegmental perfusion defects in the absence of acute pulmonary embolism suggestive of microvascular thrombosis.

## Introduction

Severe acute respiratory syndrome (SARS) caused by the coronavirus SARS-CoV-2 (COVID-19) has a high mortality due primarily to respiratory failure. Recent studies have highlighted increased thrombosis in COVID-19.^1^ Extensive microvascular thrombosis has been noted at post-mortem and high rates of pulmonary embolism (PE) diagnosed using computed tomography pulmonary angiography (CTPA),^2^ alongside lung parenchymal changes.^3^ There has been increasing interest in the patterns of pulmonary vascular involvement due to COVID-19 and concern that perfusion abnormalities may represent in-situ thrombosis that may not be appreciated on standard CTPA. The British Thoracic Imaging Society Guidelines recommend unenhanced pulmonary angiography and CTPA. This protocol facilitates lung subtraction iodine mapping (CT-LSIM) for lung perfusion, a clinically sensitive tool in PE. We report the first CT-LSIM images in COVID-19.

## Materials and methods

At our institution, 10 patients (mean age (SD) 70 (16), 40% female) with COVID-19, confirmed on reverse transcription polymerase chain reaction (RT-PCR), underwent CTPA and CT-LSIM for suspected acute PE based on clinical assessment and elevated d-dimer levels (Table 1). Analysis of CT images was approved by our institution review board.

**Table 1.** Patients’ characteristics.

Characteristic	Total (n = 10)	No pulmonary vascular changes CTPA/CT-LSIM (n = 6)	Pulmonary vascular changes CTPA/CT-LSIM (n = 4)
Age, years	70 (16)	73 (17)	68 (18)
Female, n	4	3	1
Race or ethnic group, n			
White	8	4	4
Black	1	1	0
Other ethnicity	1	1	0
Comorbidities, n			
Obesity	2	1	1
Smoker	3	2	1
Immunosuppression	2	1	1
Malignancy	1	0	1
Chronic obstructive pulmonary disease	1	1	0
Asthma	2	1	1
Ischaemic heart disease	1	0	1
Hypertension	5	2	3
Diabetes mellitus	2	1	1
Chronic kidney disease	1	0	1
Symptoms and signs around time for CTPA, n			
Tachycardia	4	2	2
Chest pain	2	1	1
Hypoxia	8	4	4
Intubation	2	1	1
Length of hospital stay, days	23 (13)	17 (16)	27 (15)
Critical care admission, n	5	2	3
Peak d-dimer 30 days prior to CTPA, ng/ml^a^	1637 (1075–18,902)	1160 (870–15,240)	8130 (2339–23,317)
Peak c-reactive protein 7 days prior to CTPA, mg/L	192 (127)	204 (84)	181 (170)
Peak Ferritin 30 days prior to CTPA, μg/L^a^	1036 (347–1928)	1089 (218–1360)	982 (342–1801)

Data are presented as mean (SD) or numbers. CTPA: computed tomography pulmonary angiography; CT-LSIM: computed tomography lung subtraction iodine mapping.

^a^Median (interquartile range).

## Results

**Figure 1.**
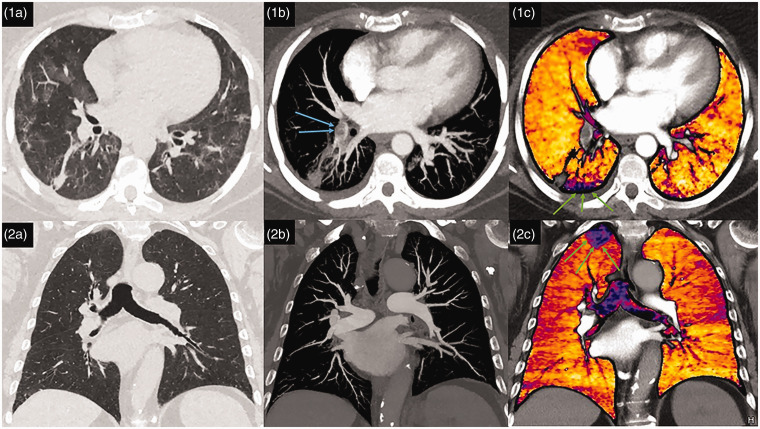
Patterns of parenchymal, vascular, and perfusion abnormalities in COVID-19. Patient 1: (a) Peripheral wedge-shaped abnormality in the right lower lobe associated with a segmental filling defect (blue arrows) on CTPA (b) corresponding to a perfusion defect (green arrows) on CT-LSIM (c). Patient 2: (a) Absence of lung parenchymal involvement and normal pulmonary vasculature on CTPA (b) with a perfusion defect in the right upper lobe on CT-LSIM (c, green arrows).

Three patients had confirmed PE on CTPA and CT-LSIM (one case is shown in Fig. 1 (1a, 1b, 1c)). Another patient had perfusion defects on CT-LSIM without visible PE where CT-LISM showed subsegmental perfusion defects without visible PE (Fig. 1 (2a, 2b, 2c)). Six patients did not have perfusion defects on CT-LSIM.

## Discussion

Distinct from classical thromboembolic PE, a high proportion of in situ pulmonary arterial thrombosis exists in COVID-19, and the pathophysiology is not fully understood.^4^ Here, we demonstrate patterns of pulmonary vascular disease in COVID-19 including (i) classical acute PE with central clot associated with lung infarction and (ii) subsegmental perfusion defects in the absence of acute PE which is perhaps suggestive of microvascular thrombosis.

CT-LSIM is potentially widely available for the assessment of lung perfusion in COVID-19. Further studies to understand the pathophysiology of pulmonary thrombotic disease in COVID-19 are required.
